# *Cirrhilabruswakanda*, a new species of fairy wrasse from mesophotic ecosystems of Zanzibar, Tanzania, Africa (Teleostei, Labridae)

**DOI:** 10.3897/zookeys.863.35580

**Published:** 2019-07-11

**Authors:** Yi-Kai Tea, Hudson T. Pinheiro, Bart Shepherd, Luiz A. Rocha

**Affiliations:** 1 School of Life and Environmental Sciences, University of Sydney, Sydney, Australia University of Sydney Sydney Australia; 2 Australian Museum Research Institute, Australian Museum, 1 William St, Sydney NSW 2010 Australia Australian Museum Research Institute, Australian Museum Sydney Australia; 3 Department of Ichthyology, California Academy of Sciences, San Francisco, CA, USA Department of Ichthyology, California Academy of Sciences San Francisco United States of America; 4 Steinhart Aquarium, California Academy of Science, San Francisco, CA, USA Steinhart Aquarium, California Academy of Science San Francisco United States of America

**Keywords:** Coral reefs, deep reefs, Indian Ocean, rebreather diving, reef fish

## Abstract

*Cirrhilabruswakanda***sp. nov.** is described on the basis of the holotype and four paratypes collected between 50 and 80m depth over low-complexity reef and rubble bottoms at the east coast of Zanzibar, Tanzania, Africa. The new species belongs to a group of fairy wrasses from the western Indian Ocean, sharing a combination of characters that include: short pelvic fins (not or barely reaching anal-fin origin); relatively unmarked dorsal and anal fins; males with a strongly lanceolate caudal fin (except in *C.rubrisquamis*); both sexes with a pair of prominent facial stripes above and below the orbit; and both sexes with prominent purple scales and osseus elements that persist, and stain purple, respectively, even in preservation. This group of fairy wrasse is part of a larger complex that includes related species from the western Pacific Ocean. In addition to meristic and morphometric comparisons, we also compare mitochondrial DNA sequence data to the aforementioned, putatively related species.

## Introduction

The labrid fish genus *Cirrhilabrus* Temminck & Schlegel, 1845 consists of small, colourful, planktivorous fishes found mostly on rubble slopes adjacent to coral reefs. Allen et al. (2015) listed 51 valid species in the genus. Eight other species have subsequently been described: *Cirrhilabrusisosceles* Tea et al., 2016, *C.hygroxerus* Allen & Hammer, 2016, *C.rubeus* Victor, 2016, *C.efatensis* Walsh et al., 2017, *C.shutmani* Tea & Gill, 2017, *C.greeni* Allen & Hammer, 2017, and *C.cyanogularis* Tea et al., 2018, bringing the valid species count to 59.

Members of this genus occur exclusively within the Indo-Pacific, attaining their highest diversity in the western Pacific Ocean and eastern Indian Ocean. In contrast, only seven nominal species have been reported from the western Indian Ocean, just slightly more than 10% of the genus. These are: *Cirrhilabrusexquisitus* Smith, 1957, *C.blatteus* Springer & Randall, 1974, *C.rubriventralis* Springer & Randall, 1974, *C.rubrisquamis* Randall & Emery, 1983, *C.sanguineus* Cornic, 1987, *C.africanus* Victor, 2016, and *C.rubeus* Victor, 2016. Of these, *C.sanguineus*, *C.blatteus*, and *C.rubrisquamis* are common only in mesophotic ecosystems, at depths greater than 40 m (Randall and Emery 1983; Springer and Randall 1974; Tea et al. 2018)

Mesophotic coral ecosystems (MCEs) characterise the deeper portions of coral reefs, found between 30 and 150 m (Rocha et al. 2018). While the number of studies conducted in MCEs of the Atlantic, Pacific, and northern Red Sea has increased in recent years (Loya et al. 2016), few researchers have investigated deep reefs of the western Indian Province. In a recent expedition organised by the California Academy of Sciences’ “Hope for Reefs” initiative, we had the opportunity to study the fish biodiversity in MCEs of Zanzibar, western Indian Ocean. While exploring deep reefs through technical rebreather diving, the authors discovered a new species of fairy wrasse belonging to the genus *Cirrhilabrus*. We herein describe *Cirrhilabruswakanda* sp. nov., the 60^th^ recognised species of the genus and the eighth species recorded from the western Indian Ocean.

## Materials and methods

Specimens of the new species were collected using hand nets while diving on mixed-gas, closed-circuit rebreathers (Hollis Prism 2). Methods of counting and measuring follow Randall and Masuda (1991). Gill raker counts follow Tea and Gill (2017) and are presented as upper (epibranchial) + lower (ceratobranchial); the angle raker is included in the second count. Data are presented as the range of all specimens examined, followed by data for the holotype in parentheses. Where counts were recorded bilaterally, both counts are given and separated from each other by a slash; the first count presented is the left count. Morphometric values are presented in Table 1, expressed as percentage of standard length. Institutional codes follow Sabaj (2016) and are as follows:

**Table 1.** Proportional measurements of type specimens of *Cirrhilabruswakanda* sp. nov. expressed as a percentage of the standard length.

	**Holotype**	**Paratypes**			
	**CAS 246395**	**CAS 246397**	**CAS 246398**	**CAS 246399**	**CAS 246396**
Sex	male	male	female	female	female
Standard length (mm)	70.3	61.3	57.4	54.3	56.8
Body depth	30.9	31.7	29.8	31.9	31.8
Body width	11.8	12.9	12.6	13.9	14.5
Head length	31.0	30.6	31.2	30.1	27.7
Snout length	8.0	8.9	7.9	8.2	7.4
Orbit diameter	6.6	8.0	7.2	9.0	7.7
Interorbital width	8.5	9.6	7.7	9.3	9.1
Upper jaw length	6.9	8.2	6.5	7.4	8.2
Caudal-peduncle depth	15.1	16.3	14.8	16.3	16.5
Caudal-peduncle length	12.8	16.5	14.1	14.9	14.8
Predorsal length	32.6	33.8	31.9	31.7	33.7
Preanal length	60.4	59.5	59.6	58.5	61.4
Prepelvic length	34.4	33.1	31.5	35.7	36.4
Dorsal-fin base	58.2	56.6	55.3	63.2	57.0
First dorsal spine	5.4	6.5	6.2	5.2	6.4
Longest dorsal spine	11.9	14.3	12.4	13.6	12.7
Longest dorsal ray	19.0	18.3	16.8	16.7	17.2
Anal-fin base	26.1	25.3	25.4	27.6	24.6
First anal spine	6.0	6.4	5.2	5.7	6.4
Second anal spine	9.1	9.3	9.3	9.4	10.1
Third anal spine	10.5	11.1	10.8	10.9	11.4
Longest anal ray	16.8	17.8	14.5	15.1	17.9
Caudal-fin length	28.2	28.6	25.4	28.0	31.6
Pectoral-fin length	19.6	21.8	20.6	18.3	20.3
Pelvic spine length	11.2	12.1	11.7	11.0	11.3
Pelvic fin length	18.0	17.9	16.2	15.5	18.8

**CAS**California Academy of Sciences.

DNA extraction and PCR amplification of the mitochondrial cytochrome c oxidase subunit I (COI) were performed following protocols detailed in Weigt et al. (2012). Forward and reverse contigs were aligned and trimmed separately using Geneious Prime 2019.1.1. (Biomatters, Auckland, New Zealand). Uncorrected pairwise distances for the COI marker were calculated in Geneious Prime. We compared the DNA sequences from four specimens of the new species to putatively related species of *Cirrhilabrus* with publicly available sequence data in GenBank (*Cirrhilabrussanguineus*: MH780162; *Cirrhilabrusrubrisquamis*: MH780161; *Cirrhilabrusblatteus*: MF123821).

## Taxonomy

### Cirrhilabrus wakanda sp. nov.

Taxon classificationAnimaliaPerciformesLabridae

http://zoobank.org/2E9018A1-A98F-4F8C-AEA9-89D18BC69162

**Figure 1.**
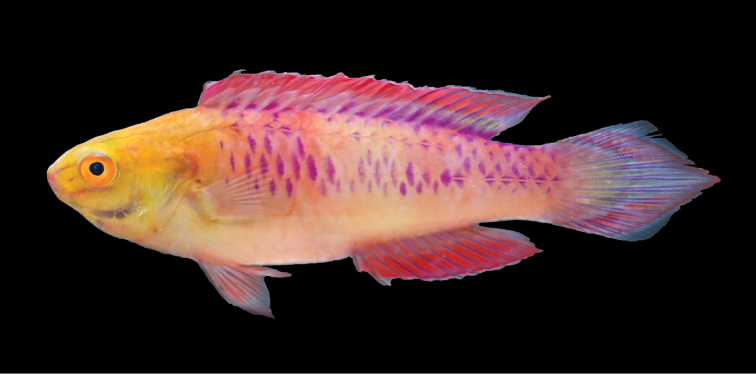
*Cirrhilabruswakanda* sp. nov., freshly euthanized male holotype (CAS 246395), 70.3 mm SL, male, collected at a depth of 75 m, east coast of Zanzibar, Africa (above). Note the pair of facial stripes above and below orbit. Photograph by H.T. Pinheiro and B. Shepherd.

**Figure 2.**
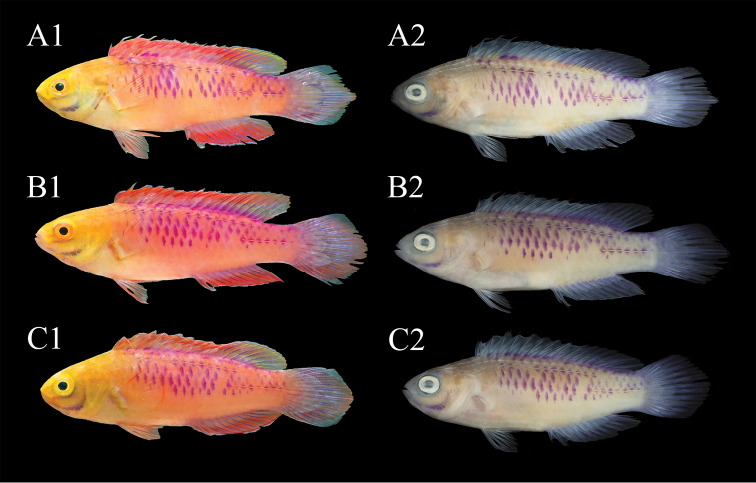
Paratypes of *Cirrhilabruswakanda* sp. nov., not to scale **A1** CAS 246397, 61.3 mm SL, male, freshly euthanized **A2** CAS 246397, male in preservation **B1** CAS 246398, 57.38 mm SL, female, freshly euthanized **B2** CAS 246398, female in preservation **C1** CAS 246399, 54.32 mm SL, female, freshly euthanized **C2** CAS 246399, female in preservation. Photographs by H.T. Pinheiro and B. Shepherd (**A1, B1, C1**), and J. Fong (**A2, B2, C2**).

**Figure 3.**
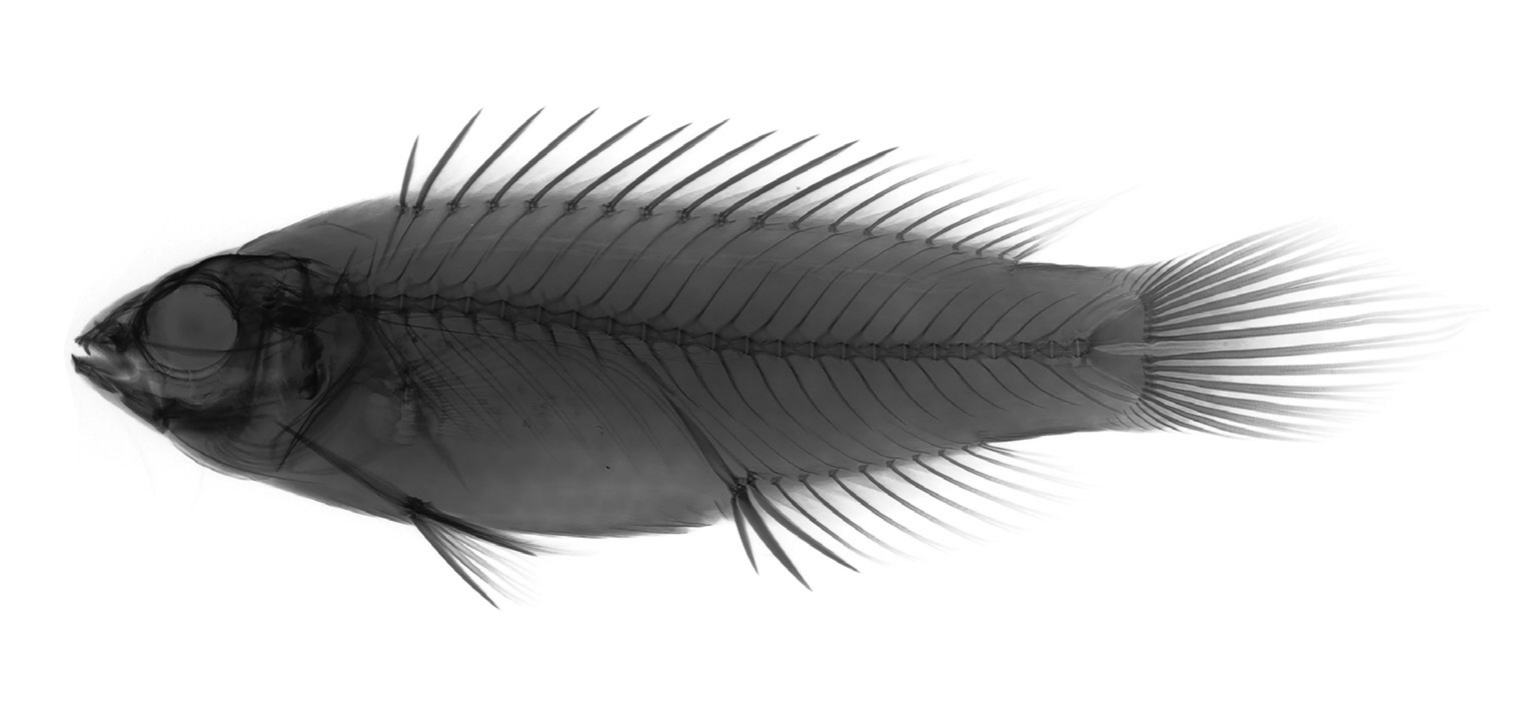
*Cirrhilabruswakanda* sp. nov., CAS 246395, 70.3 mm SL, male holotype, x-ray. Radiograph by J. Fong.

**Figure 4.**
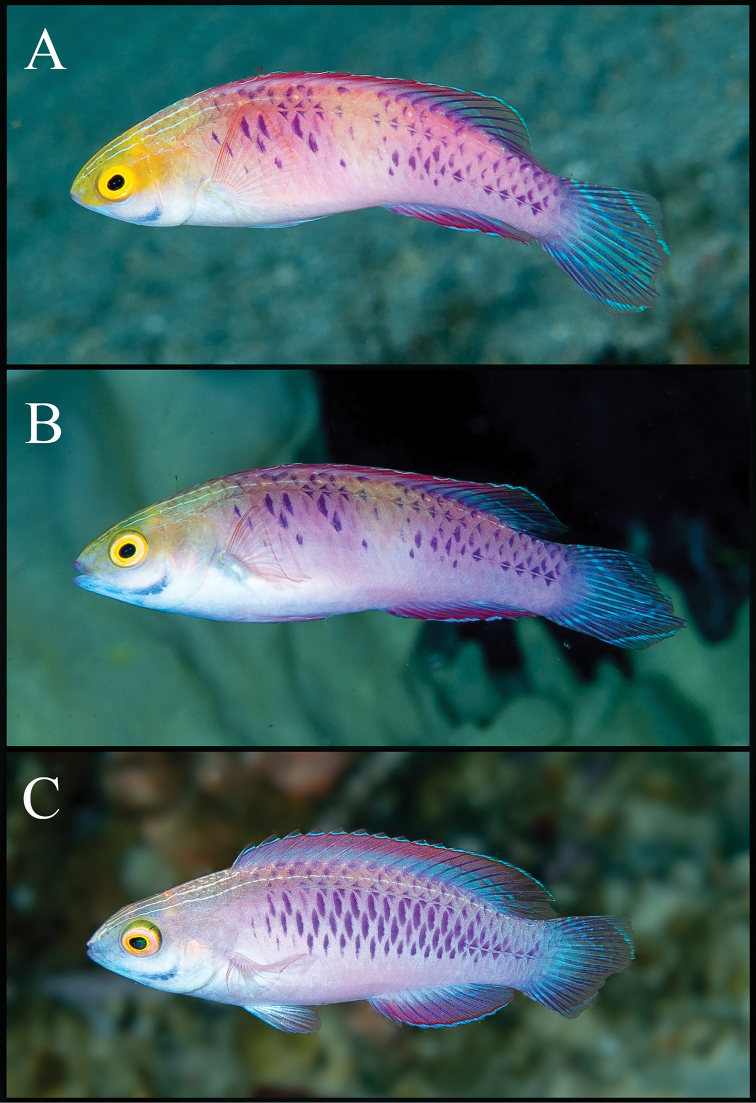
*Cirrhilabruswakanda* sp. nov., in situ photographs at 75 m depth, in the east coast of Zanzibar, Tanzania, Africa. Specimens not retained. Note intensity of yellow on the heads of males (**A**), transitioning males (**B**), and females (**C**). Photographs by L.A. Rocha.

**Figure 5.**
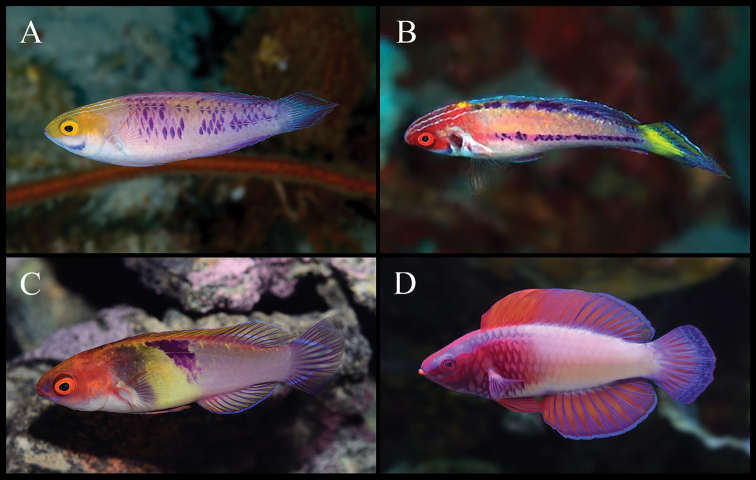
A selection of *Cirrhilabrus* species from the western Indian Ocean group of the *Cirrhilabrusjordani* complex **A***Cirrhilabruswakanda* sp. nov., in situ photograph from the east coast of Zanzibar, Africa **B***Cirrhilabrusblatteus*, *in situ* photograph from the Red Sea, off the coast of Eilat; **C**: *Cirrhilabrussanguineus*, aquarium photograph of a specimen from Mauritius **D***Cirrhilabrusrubrisquamis*, aquarium photograph of a specimen from the Maldives. Photographs by L.A. Rocha (**A**); E. Brokovich (**B**), and Y.K. Tea (**C, D**).

**Figure 6.**
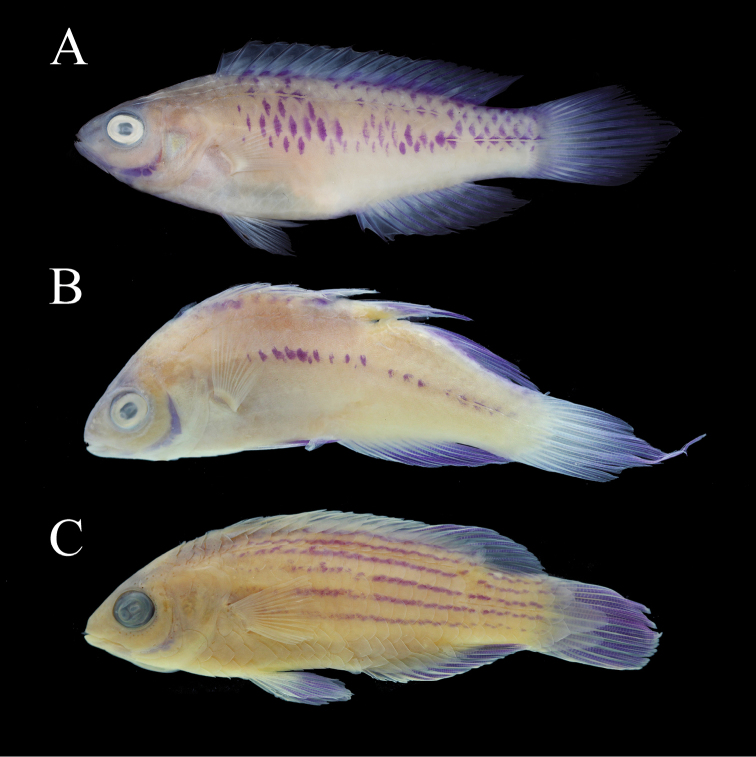
A selection of *Cirrhilabrus* species in preservation showing the purple staining qualities. Not to scale **A***Cirrhilabruswakanda* sp. nov., 70.3 mm SL, male holotype, CAS 246395 **B***Cirrhilabrusblatteus*, 65.1 mm SL, male, CAS 235080 **C***Cirrhilabrusearlei*, 56.5 mm SL, male paratype, CAS 213114. Photographs by L.A. Rocha (**A**) and B.W. Frable (**B, C**).

Vibranium fairy wrasse Figures 1 , 2 , 3 , 4 , 5A , 6A ; Table 1

#### Holotype.

CAS 246395 (field code: HTP 900), 70.3 mm SL male, GenBank MN010585, east coast of Zanzibar, Tanzania, Africa (GPS coordinates: 6°10'30"S; 39°32'28"E), 75 m, collected by H.T. Pinheiro, B. Shepherd, and L.A. Rocha, 14 December 2018; Figure 1.

#### Paratypes.

CAS 246396 (HTP 883), 56.8 mm SL female, GenBank MN010586, east coast of Zanzibar, Tanzania, Africa, 70 m, 07 December 2018; CAS 246397 (HTP 901), 61.3 mm SL male, GenBank MN010587, east coast of Zanzibar, Tanzania, Africa, 75 m, 14 December 2018; Figure 2 (A1, A2); CAS 246398 (HTP 902), 57.4 mm SL female, GenBank MN010588, east coast of Zanzibar, Tanzania, Africa, 75 m, 14 December 2018; Figure 2 (B1, B2); CAS 246399 (HTP 903), 54.3 mm SL female, east coast of Zanzibar, Tanzania, Africa, 75 m, 14 December 2018; Figure 2 (C1, C2). All type specimens collected by H.T. Pinheiro, B. Shepherd, and L.A. Rocha.

#### Diagnosis.

*Cirrhilabruswakanda* shares similar meristic characters to other members of this genus. However, it is readily distinguished from all other *Cirrhilabrus* in having the following combination of colouration and morphological characters: caudal fin strongly lanceolate in males; both sexes with a series of purple scales (in life and in preservation) arranged in a chain-link pattern across dorsal two-thirds of body.

#### Description.

Dorsal-fin rays XI,9; anal-fin rays III,9; dorsal and anal-fin soft rays branched except first ray unbranched in two individuals; last dorsal and anal-fin ray branched to base; pectoral-fin rays 14–15 (15/15), upper two unbranched; pelvic-fin rays I,5; principal caudal-fin rays 7+6, uppermost and lowermost unbranched; upper procurrent caudal-fin rays 6, lower procurrent caudal-fin rays 6; lateral line interrupted, with dorsoanterior series of pored scales 16–19 (17/17) and midlateral posterior peduncular series 8–9 (9/9); scales above lateral line to origin of dorsal fin 2; scales below lateral line to origin of anal fin 6; median predorsal scales 4–5 (4); median prepelvic scales 5; rows of scales on cheek 2; circumpeduncular scales 15–16 (15); gill rakers 8–9 (8) + 8–9 (8) = 16–18 (16); pseudobranchial filaments 8–10; vertebrae 9+16; epineurals 13 (Figure 3).

Body moderately elongate and compressed, depth 3.1–3.4 (3.2) in SL, width 2.1–2.6 (2.6) in depth; head length 3.2–3.6 (3.2) in SL; snout pointed, its length 3.4–3.9 (3.9) in HL; orbit diameter 3.6–4.7 (4.7) in HL; depth of caudal peduncle 1.7–2.1 (2.1) in HL. Mouth small, terminal, and oblique, with maxilla almost reaching vertical at front edge of orbit; dentition typical of genus with three pairs of canine teeth present anteriorly at side of upper jaw, first forward-projecting, next two strongly recurved and outcurved, third longest; an irregular row of very small conical teeth medial to upper canines; lower jaw with a single stout pair of canines anteriorly which protrude obliquely outward and are slightly lateral to medial pair of upper jaw; no teeth on roof of mouth.

Posterior margin of preoperculum with 30–32 (32) very fine serrated; margins of posterior and ventral edges of preoperculum free to about level of middle pupil. Anterior nostril in short membranous tube, located nearer to orbit than snout tip; posterior nostril larger, roughly ovoid to rectangular, located just medial and anterior to upper edge of eye. Scales cycloid; head scaled except snout and interorbital space; four large scales on opercle; a broad naked zone on membranous edge of preopercle; a row of large, elongate, pointed scales along base of dorsal fin, one per element, scales progressively shorter posteriorly on soft portion of fin; anal fin with a similar basal row of scales; last pored scale of lateral line (posterior to hypural plate) enlarged and pointed; one scale above and below last pored scale also enlarged; a horizontal series of greatly enlarged scales extend two-thirds distance to central posterior margin of caudal fin; pectoral fins naked except for a few small scales at extreme base; a single large scale at base of each pelvic fin, about three-fourths length of pelvic spine.

Origin of dorsal fin above third lateral-line scale, predorsal length 3.0–3.2 (3.1) in SL; first 1–4 dorsal-fin spines progressively longer, fifth to sixth subequal, eighth to tenth longest, 2.1–2.6 (2.6) in HL; interspinous membranes of dorsal fin in males extend beyond dorsal-fin spines, with each membrane extending in a pointed filament beyond spine; fifth dorsal-fin soft ray longest, 1.6–1.9 (1.6) in HL, remaining rays progressively shorter; origin of anal fin below base of ninth dorsal-fin spine; third anal-fin spine longest, 2.4–3.0 (3.0) in HL; interspinous membranes of anal fin extended as on dorsal fin; anal-fin soft rays relatively uniform in length, sixth longest, 1.5–2.1 (1.8) in HL; dorsal and anal-fin rays barely reaching caudal-fin base; caudal fin of males lanceolate; pectoral fins short, reaching vertical between bases of fifth or sixth dorsal-fin spines, longest ray 1.4–1.6 (1.6) in HL; origin of pelvic fins below lower base of pectoral fins; pelvic fins short, not reaching past anal fin origin, longest ray 1.5–1.9 (1.7) in HL.

#### Colouration of males in life.

Based on colour photographs and specimens when freshly dead, and field photos of live individuals (Figures 1; 2A1; 4A; 4B; 5A): head ochreous yellow; lower part of head whitish to pale pink (yellowish when freshly dead), purple stripe present from mid-upper lip to mid-upper edge of orbit; second stripe of similar colour present from lower edge of maxilla to mid-lower edge of orbit; interorbital and upper part of snout yellowish, with a series of very fine white stripes; preoperculum prominently purple on outer edge; iris bright yellow, greenish on the upper edge, with orange ring around pupil; body pale mauve to purplish-pink, with a faint region of paler yellowish-pink below middle part of dorsal fin; body with a network of dark purple scales arranged in a chain-link pattern from just after dorsal fin origin to edge of caudal peduncle, absent from lower third of body; dorsal-fin bluish-purple, bright fuchsia on distal half; posterior dorsal fin yellowish hyaline with a faint blue medial band, sometimes broken into spots; distal edge of dorsal fin narrowly bright blue; caudal fin bluish hyaline with a pair of concentric bright blue chevrons converging at lanceolatus terminus; coloured portion of chevron marking bright fuchsia to magenta; anal-fin similar to dorsal fin, distal edge narrowly bright blue; pelvic fins hyaline to translucent magenta; pectoral fins pinkish hyaline.

#### Colouration of females and juveniles in life.

Similar to males described above. Head and body more subdued in colouration, pinkish-purple to lilac (Figure 4C), deepening to yellow post mortem (Figures 2B1; 2C1).

#### Colouration in preservative.

(Figures 2A2; 2B2; 2C2; 6A): head and body pale tan; fine white stripes on interorbital and nape remain; infraorbitals, frontals, and pre-maxilla weakly purple; preoperculum, dentary, angular, and articular bones strongly purple; scales in chain-link formation deep purple; median fins translucent, except rays weakly purple; pelvic and pectoral fins translucent hyaline.

#### Etymology.

The specific epithet refers to the fictional East African nation of Wakanda, home of the superhero Black Panther, as is the case for the new species, which has remained hidden from the world for a long time. To be treated as a noun in apposition. The common name refers to the fictional metal vibranium, a rare substance found on Wakanda that is woven into Black Panther’s suit. The purple chain-link scale pattern of the new species is reminiscent of this detail.

#### Distribution and habitat.

*Cirrhilabruswakanda* is presently known only from the east coast of Zanzibar, Tanzania. The species inhabits deep shelves consisting of small patch reefs dominated by rhodolith and sponge beds, at depths between 50 and 80 m.

#### Comparisons.

Pairwise comparison of mitochondrial sequence data suggests that *Cirrhilabruswakanda* is most closely related to *C.rubrisquamis* Randall & Emery (1983), differing by 0.6% in mitochondrial COI (uncorrected pairwise distance). Such marginal differences in sequence data between closely related sister species is not uncommon in *Cirrhilabrus*, even when stark morphological differences are present (Tea et al., 2016; Victor, 2016; Allen & Hammer, 2017). It also appears to be closely related to *C.blatteus* Springer & Randall (1974) (1.9% difference in COI) and *C.sanguineus* Cornic (1987) (1.5% difference in COI). These four species share the following character combination: short pelvic fins (not or barely reaching anal-fin origin); relatively unmarked dorsal and anal fins; males with a strongly lanceolate caudal fin (except in *C.rubrisquamis*); both sexes with a pair of prominent facial stripes above and below the orbit; and both sexes with prominent purple scales and osseus elements that persist, and stain purple, respectively, even in preservation.

In *Cirrhilabruswakanda* the purple scale pattern presents as a scattered, chain-link motif (Figure 1; 2; 4; 5A). In the other related species, the purple scales are manifested as: two rows dorsally and laterally in *C.blatteus* (Figure 5B); an oblique mid-dorsal saddle in *C.sanguineus* (Figure 5C); a crosshatch network anteriorly in *C.rubrisquamis*(Figure 5D). Aside from details in live colouration, *Cirrhilabruswakanda* differs from: *C.blatteus* in having a higher number of pored lateral line scales (24–28 vs. 21–24); *C.sanguineus* in having one fewer median prepelvic scale (5 vs. 6) and fewer pseudobranchial filaments (8–10 vs. 11), and further from *C.rubrisquamis* in having a lanceolate caudal fin.

The four species are part of a larger complex of fairy wrasses that includes five other species from the western Pacific Ocean: *Cirrhilabrusjordani*, *C.earlei*, *C.roseafascia*, *C.lanceolatus*, and *C.shutmani*. Together, these nine species form the *Cirrhilabrusjordani* complex. Previous morphological and molecular studies have also shown support for this grouping (Tea and Gill 2017; Tea et al. 2018).

#### Remarks.

*Cirrhilabruswakanda* possess several osseus elements and fin rays that stain naturally purple in ethanol (Figure 6A). Only a handful of other *Cirrhilabrus* share this character. Springer and Randall (1974) first noted this occurrence in *Cirrhilabrusblatteus* (Figure 6B). Subsequently, Randall (1995) made note of its reoccurrence in *Cirrhilabrusrubrisquamis* and *Cirrhilabrussanguineus*. Tea et al. (2018) expanded this list to include *Cirrhilabrusearlei* (Figure 6C). Incidentally, these species are all closely related members of the *jordani* complex, with *C.wakanda*, *C.rubrisquamis*, *C.sanguineus* and *C.blatteus* occurring in the western Indian Ocean, and *C.earlei* occurring in the western Pacific Ocean. However, since the purple post-preservation staining is not found in the other Pacific Ocean species (*C.jordani*, *C.shutmani*, *C.roseafascia*, and *C.lanceolatus*), the distribution of this character within the *jordani* complex sensu lato is paraphyletic and is therefore not synapomorphic for this group of fairy wrasses.

#### Material examined.

*Cirrhilabrusblatteus* – Red Sea, off Saudi Arabia: CAS 235080, 56.2 mm SL; 63.4 mm SL; 65.1 mm SL; *Cirrhilabrusearlei* – Palau: CAS 213114, 56.5 mm SL.

## Supplementary Material

XML Treatment for Cirrhilabrus wakanda
